# The Importance of Testing the Ocular Muscles

**Published:** 1908-02

**Authors:** R. B. Ridley

**Affiliations:** Clinical Professor of Eye, Ear, Nose and Throat, Atlanta School of Medicine


### THE IMPORTANCE OF TESTING THE OCULAR MUSCLES.

BY R. B. RIDLEY, JR., M. D., CLINICAL PROFESSOR OF EYE, EAR, NOSE AND THROAT, ATLANTA SCHOOL OF MEDICINE.

---

In recent years, a great deal has been written upon the subject of errors of refraction and their treatment, but, comparatively, little has been said about the condition of the muscles of the eyes in connection with these errors. To my mind, these two subjects are so closely allied that, when speaking of one, I invariably think of the other.

When we think of the wonderful amount of work which these little muscles, almost the smallest in the human anatomy, are called upon to perform, we can scarcely wonder that so many unpleasant symptoms arise from any imperfection of them. In this paper, I only intend to discuss the latent muscular weaknesses, with their treatment, and at some future time to take up the subject of Strabismus or Squint.

In an emmetrope, who has perfect muscular balance, the eyes are able to "fix" an object properly and the retinal images formed upon the fovea of each eye are fused into one and as a result the brain sees only one, but when such is not the case and one eye turns in some other way, contrary to the direction of the eye which "fixes," one image is made upon the fovea of the "fixing eye," while another image is formed upon some point of the retina of the deviating eye distant from the fovea; as a consequence of this, the brain takes cognizance of two objects and diplopia, in some form, results.

One image, the one made upon the retina of the "fixing" eye, is seen distinctly by the brain, while the image made upon the retina of the deviating eye is blurred, or is seen by the brain indistinctly; known, respectively, as the true and false image.

In cases of latent muscular weakness, this false image may be done away with by stimulation of the weakened muscle, in order to draw the deviating eye in such a position that it may also "fix" the object and have the impression made upon its fovea at the same time as in the "fixing" eye. In strabismus, this stimulation cannot be accomplished on account of the decided weakness of the muscle at fault, and the person so afflicted will continue to have diplopia, unless the trouble is corected by lenses properly prescribed, or until he learns to ignore the false image, which, in time, they are generally enabled to do, though greatly to the injury of the deviating eye, as amblyopia exanopsia is almost certain to result.

Heterophoria is divided into several classes, though I will only give here those which concern me in my present paper; Hyperphoria, sometimes erroneously termed hypophoria, or a tendency of one eye to deviate upward; esophoria a tendency of the visual axes to turn inward; exophoria, a tendency of the visual axes to deviate outward.

The above muscle troubles are the ones most commonly found and the ones which give the most severe symptoms; they are, therefore, the most important to the oculist.

Symptoms: The symptoms which arise from muscle insufficiences are many and in a great number of cases, quite severe, causing neuralgia, sick headache, nausea and often a general nervous derangement of the entire system, when the eyes are entirely normal in other respects.

Some prominent oculists have even gone so far as to state that muscular insufficiences of the ocular muscles are, in some instances, the direct cause of epilepsy and other serious mental derangements.

Headaches, so often complained of by women after a day's shopping; those complained of after a night at the opera, or those who do a great amount of near work, as seamstresses, book-keepers and others who have like vocations, can often be relieved by the careful oculist, when the efforts of the general practitioner have failed.

Any one can tell whether a person has strabismus or not, but it remains with the oculist to determine whether a person has latent muscle trouble and it is impossible for him to determine without a careful test of each muscle separately. The oculist who would sit a patient down by his trial case, put on a plus or minus lens and prescribe for the error of refraction which may be present, without testing the patient's ocular muscles, both static and dynamic for near and distant work, is guilty of a gross injustice to the patient, and does not deserve to be classed as an oculist.

The treatment for these muscular insufficiences are both operative and non-operative. The operative methods consist of tenotomies; the non-operative methods consist of exercise with prisms, full correction of refractive errors, if any be present, and constant wearing of prisms in connection with this correction.

The operative treatment of this muscular trouble should not be attempted until all other methods have been tried and failed, for often an operation of this character will result in an over-correction without benefit to the patient.

It has been my privilege to have a few patients come to me whose error of refraction has been corrected by proper lenses, prescribed by some other oculist, but whose symptoms have not been relieved entirely. By testing the muscles of these patients, I have found that most of them have some ocular weakness, and which, when strengthened by exercise, are greatly relieved. After testing the muscles of these patients, I have asked if this had been done before, and everyone, with one exception, has answered in the negative. These tests of the ocular muscles, to my mind, are almost as important as correcting a high degree of myopia or rypermetropia.

The only reason I can attribute to an oculist for not examining the muscles, is that it takes time to do so,—but one pleased patient is worth a dozen displeased patients.

